# Validity and reliability of the Neonatal Infant Acute Pain Assessment Scale (NIAPAS) in Turkish: prospective study

**DOI:** 10.1590/1516-3180.2020.0721.R1.23122020

**Published:** 2021-08-02

**Authors:** Bahar Nur Kanbur, Birsen Mutlu, Özgül Salihoğlu

**Affiliations:** I RN, PhD. Assistant Professor, Department of Nursing, Istanbul Gelisim University, Istanbul, Turkey; II RN, PhD. Associate Professor, Pediatric Nursing Department, Florence Nightingale Nursing Faculty, Istanbul University-Cerrahpaşa, Istanbul, Turkey; III MD, PhD. Professor, Neonatal Intensive Care Clinic, Istanbul Bakırköy Dr. Sadi Konuk Training and Research Hospital, Istanbul, Turkey.

**Keywords:** Infant, newborn, Pain, Acute pain, Pain measurement, NIAPAS, Scale, Reliability, Validity

## Abstract

**BACKGROUND:**

Using pain scales helps nurses in making early diagnoses and in assessing and managing pain symptoms and findings when developing a nursing care plan.

**OBJECTIVE:**

To determine the validity and reliability of the Turkish form of the Neonatal Infant Acute Pain Assessment Scale (NIAPAS).

**DESIGN AND SETTING:**

Prospective study conducted in Istanbul Bakırköy Dr. Sadi Konuk Training and Research Hospital, Istanbul, Turkey.

**METHODS:**

145 newborns in the 26^th^ to 42^nd^ gestational weeks that were receiving treatment and care in the neonatal intensive care unit were included in this study. A total of 1740 pain assessments were made by two independent observers on these 145 newborns. The research data was collected using a newborn description form, NIAPAS and the Neonatal Infant Pain Scale (NIPS).

**RESULTS:**

The scope validity index of NIAPAS was found to be between 0.90 and 1.00 and its Cronbach’s alpha coefficient was 0.914. Correlations between characteristics and total scores (r = 0.20-0.82) were found to be sufficiently high. In an assessment on concurrency validity, there was a strong positive relationship between NIAPAS and NIPS scores (r = 0.73-0.82; P < 0.000). From kappa analysis (0.73-0.99) and intraclass correlation (r = 0.75-0.96), it was determined that there was concordance between the observers.

**CONCLUSION:**

NIAPAS was found to be a valid and reliable scale for evaluating acute pain in newborns.

## INTRODUCTION

Newborns are subject to many painful interventions during their stay in neonatal intensive care units (NICUs).^1^ Studies have shown that an average of 115 operations are performed in NICUs within a two-week period and that 75% of these operations are painful interventions.^2^^,^^3^ Newborns are subjected to an average of 7.6-14 painful interventions per day in the first 14 days of their hospitalization.^2^^,^^4^

Until recently, it was considered that newborns could not feel pain because their nervous system is not well-developed and myelinization has not yet been completed.^5^-^7^ However, new studies have shown that pain is perceived before the 24^th^ intrauterine gestational week^8^ and infants give behavioral, physiological and hormonal responses to painful stimuli during the 25^th^ to 36^th^ gestational weeks.^9^ The pain received might result in physiological imbalances in newborns and abnormalities in brain development or response to stress over the short or long term.^10^ Untreated pain could have a negative impact on communication between the family and the infant and could also lead to emotional and psychosomatic problems in the future.^11^^,^^12^

Accurate assessment of the pain is the first step in enabling effective pain management.^13^ The most important problem encountered in understanding pain in newborns is the difficulty in diagnosing pain.^14^ Because newborn infants are unable to verbally express pain, any pain assessment is based on the ability of other people to identify pain symptoms.^4^

The American Academy of Pediatrics has recommended that clinical personnel should routinely perform reliable and valid measurements on newborns.^15^ In NICUs, pain should be assessed by healthcare professionals who have been trained to identif and interpret pain and to consider factors that impact on newborns’ perception of pain and response to pain.^16^ Although significant progress in newborn pain management has been made over the last 20 years, pain is still not sufficiently diagnosed among newborns born in clinics, and attempts to reduce pain are only made infrequently.^17^

Many scales are available to assess pain in NICUs. However, only a limited number of scales for assessing acute pain in preterm and term babies are available.

## OBJECTIVE

The objective of this study was to determine the validity and reliability of the Turkish form of the Neonatal Infant Acute Pain Assessment Scale (NIAPAS).

## METHODS

### Type of study

This was a prospective study conducted in Istanbul Bakırköy Dr. Sadi Konuk Training and Research Hospital, Istanbul, Turkey.

### Population and sample

The population of the study comprised babies who were followed up in the NICU, at gestational ages of 28-42 weeks and who had not been administered drugs with analgesic, sedative or muscle relaxant impacts that could affect the pain or behavior of the infant. In addition, these babies did not have hyperbilirubinemia, congenital anomalies or neurological diseases and did not undergo surgical interventions.

The sample size for this study was determined in accordance with the principle of taking the sample size to be at least five times the number of scale items, in validity and reliability assessments on study methodologies.^18^ Based on this principle, 145 infants were included in this study.

### Data collection tools

Three data collection tools were used in this study, as follows:

*Newborn description form*: This consisted of items describing the characteristics of the infant: gender, gestational age, birth weight, Apgar score, postnatal age, respiration mode and diagnosis.

*NIAPAS:* The NIAPAS scale was developed in Finland in 2013, by Pölkki et al.^19^ It differs from other scales in that it assesses acute pain in babies. It was developed based on feedback from specialist nurses within neonatal intensive care and in close cooperation with other nurses. The NIAPAS scale assesses acute pain through five behavioral and three physiological indicators that are exhibited as contextual factors in the gestational week. These indicators are rated on a scale of 2, 3 or 4 points (0,1; or 0,1,2; or 0,1,2,3), with a potential total of 18 points. Facial expressions, muscle strain, alertness, reaction to action, heart rate, respiration and oxygen saturation are considered in assessing each newborn. Painful operations on newborns are assessed in three stages: for one minute before the painful operation, for the duration of the operation and for one minute following the operation. The pain on the scale is considered to be mild when the score is 0-5, medium when it is 6-9 and sharp when it is 10-18. 

*Neonatal Infant Pain Scale (NIPS)*: This was developed in 1993, by Lawrence et al.^20^ It is used to assess pain caused by interventional operations in preterm and term newborns, from their behavioral and physiological symptoms. The symptoms assessed are facial expression, crying, respiration mode, movement of arms and legs and alertness. The total score is in the range of 0-7.

### Data collection

Research data were collected through observation. Heel-prick blood drawing was chosen as the painful intervention. The infants’ response to pain and stress was video recorded by the investigator (BNK), for one minute before the intervention, for the duration of the intervention and for one minute following the intervention.

Each baby was evaluated for pain by two independent observers other than this investigator, by watching video recordings. The observers watched the video recordings separately and scored them independently in accordance with the scale. One of the observers who made this assessment had had 10 years and the other had had 11 years of neonatal intensive care nursing experience. Training on scale and scale assessment before the assessment was provided to the nurses. 

### Assessment of the data

The research data were analyzed using the Statistical Package for the Social Sciences (SPSS) 22 for Windows (IBM, Armonk, New York, United States). Descriptive statistics on numbers, percentages and arithmetic means were used in assessing the study data. The translation and back-translation method was used to assess language validity. The scope validity index was calculated to determine the content validity. The concordance of the specialist opinions was assessed by means of Kendall’s W concordance coefficient. Correlations of total item scores and Cronbach’s alpha coefficients were calculated to determine the reliability of the scale. Kappa analysis was conducted to assess the consistency between the observers and an inter-scale correlation test was conducted to assess concurrency validity.

### Ethical aspects of the research

Permission was received from Pölkki et al.,^19^ who developed the scale, to conduct assessments on the validity and reliability of the scale for use in the Turkish language on September 1, 2019. Approval was obtained from the Clinical Trials Ethics Committee of Istanbul Bakırköy Dr. Sadi Konuk Training and Research Hospital (Ethics Committee Decision No: 2020-03-20). Before collecting the research data, consent was obtained from the parents of the infants who matched the sample criteria.

## RESULTS

The infants included in the research had a mean gestational age of 33.30 ± 4.26 weeks, mean body weight of 2188 ± 1129.936 grams and mean postnatal age of 9.21 ± 7.612 days. The infants’ first-minute mean Apgar score was determined as 6.37 ± 0.941 and the fifth-minute mean Apgar score as 8.01 ± 0.764. Certain characteristics of the infants included in the research are presented in **Table 1**.

**Table 1.** Demographics of the infants included in the study (n = 145)

Infant characteristics	Number (n)	Percentage (%)
**Gender**		
Female	64	44.1
Male	81	55.9
**Gestational age**		
Less than 28 weeks	18	12.4
28-31 weeks and 6 days	37	25.5
32-36 weeks and 6 days	41	28.3
37 weeks or above	49	33.8
**Birth weight**		
Less than 1000 g	25	17.2
1000-1999 g	49	33.8
2000-2999 g	34	23.4
3000 g or higher	37	25.5
**Respiration mode**		
Connected to mechanical ventilation	32	22.1
Continuous positive airway pressure	42	29.0
Spontaneous respiration	71	49.0
**Nutrition mode**		
TPN	47	32.4
NGS/OGS	54	37.2
Oral	44	30.3
**Diagnosis**		
Preterm and low birth weight	85	58.6
Mild asphyxia	10	6.9
TTN	20	13.8
Pneumonia	13	9.0
Polycythemia	3	2.1
Hypoglycemia	14	9.7

TPN = total parenteral nutrition; NGS = Nasogastric tube; OGS = Orogastric tube; TTN = Transient tachypnea of newborn.

### Examining the validity of the scale

#### Language validity

The language validity of the scale was assessed in accordance with prescriptions in the literature.^21^ For this, one academic staff member who was a specialist in pediatric nursing and fluent in both English and Turkish and one professional interpreter translated the scale from English to Turkish. The scale translated into Turkish was then edited by the investigator by comparing the two translations. The Turkish form thus created was back-translated into English by two academic staff members who were specialists in pediatric nursing and fluent in both languages, and who had not previously seen the original English version of the scale. The expressions used in the back-translation were compared with those in the original scale and, from this analysis to ensure language equivalence, the final version of the Turkish form of the scale was created.

#### Content/scope validity

Content validity is used to assess whether a scale and each item on this scale measure the concept that needs to be measured, and whether these contain concepts differing from or other than the concept that needs to be measured. Opinions are received from relevant specialists in order to achieve this. The specialist group is recommended to consist of a minimum of three and a maximum of 20 persons. The scale is then remodeled in accordance with the suggestions and critiques provided by the specialists.^22^

To evaluate the content/scope validity in the present study, opinions were received from 10 specialists, consisting of eight academic staff members who were specialists in pediatric nursing, one academic staff member who was a specialist in gynecology and one neonatal physician who was a specialist in neonatology. These specialists assessed each expression on the scale as 1- “I agree”, 2- “The item should be slightly reviewed”, 3- “The item should be significantly reviewed” or 4- “I disagree with the item”. Subsequently, the scale items were rearranged in accordance with the recommendations of the specialists. The scope validity ratios and the scope validity index (SVI) were obtained for each item on the scale, after implementation of these rearrangements. The SVI values of the scale items were in the range 0.90-1.00.

The concordance level of the specialist opinions was analyzed by means of the nonparametric Kendall’s W test (Kendall’s W = 0.969; P = 0.000).

#### Concurrency validity

NIPS was used as the concurrency form in this study. Two observers made three assessments: one before, one during and one after the operation. The relationship between the scores given by the observers on the NIAPAS and NIPS scales are presented in **Table 2**. There was a strong positive relationship between the correlation values from Observer 1 (r = 0.78-0.82; P = 0.000) and the correlation values from Observer 2 (r = 0.73-0.80; P = 0.000). The correlation values between Observer 1 and Observer 2 were 0.87 before the operation, 0.91 during the operation and 0.94 after the operation, i.e. there was high correlation between the observers.

**Table 2.** Relationship between the Neonatal Infant Acute Pain Assessment Scale (NIAPAS) and the Neonatal Infant Pain Scale (NIPS)

Observers	Pearson correlation coefficient (r)		
	Before operation	During operation	After operation
Observer 1	0.78	0.82	0.79
Observer 2	0.73	0.74	0.80
	0.87	0.91	0.94

Note: Pearson’s correlation coefficient (r) was used to analyze the correlation between the scores received by newborns using NIAPAS and NIPS before, during and after the intervention. P < 0.001.

### Examining the reliability of the scale

#### Internal consistency

Cronbach’s alpha coefficient was calculated to assess the internal consistency of the scale. According to the assessment results from the observers, the Cronbach’s alpha values were in the range 0.930-0.971 for Observer 1 and in the range 0.913-0.972 for Observer 2. The study’s general Cronbach’s alpha value was found to be 0.914 (**Table 3**).

**Table 3.** Score averages and Cronbach’s alpha reliability coefficient values of the Neonatal Infant Acute Pain Assessment Scale (NIAPAS)

Observers	Before operation		During operation		After operation	
	Mean score	Cronbach’s alpha	Mean score	Cronbach’s alpha	Mean score	Cronbach’s alpha
Observer 1	2.159	0.930	10.979	0.954	4.751	0.971
Observer 2	2.110	0.913	10.682	0.955	4.675	0.972

Note: The internal consistency of the scale was calculated using Cronbach’s alpha.

Total item score reliability was examined as another method for assessing the internal consistency of the scale. The total item score correlations for the scale before the operation were found to be in the range 0.20-0.77 for Observer 1 and in the range 0.36-0.78 for Observer 2. The total item score correlations during the operation were in the range 0.30-0.82 for Observer 1 and in the range 0.30-0.80 for Observer 2. After the operation, they varied in the range 0.22-0.76 for Observer 1 and in the range 0.25-0.71 for Observer 2 (**Table 4**).

**Table 4.** Total item score correlation values of the Neonatal Infant Acute Pain Assessment Scale (NIAPAS)

Characteristics	Observer 1			Observer 3		
	Before operation	During operation	After operation	Before operation	During operation	After operation
Gestational age	0.46	0.32	0.36	0.36	0.30	0.37
Alertness	0.77	0.82	0.76	0.77	0.80	0.71
Facial expressions	0.76	0.71	0.76	0.78	0.77	0.70
Crying	0.63	0.77	0.76	0.70	0.78	0.68
Muscle tension	0.65	0.60	0.70	0.61	0.56	0.50
Breathing	0.54	0.58	0.46	0.60	0.58	0.46
Reaction to handling	0.30	0.66	0.68	0.55	0.63	0.65
Heart rate	0.30	0.72	0.46	0.60	0.58	0.46
SaO_2_	0.20	0.48	0.22	0.38	051	0.25

Note: Total item correlation was calculated using Pearson’s correlation coefficient (r) between the score achieved from each item and the mean total score. SaO_2_= Oxygen saturation.

### Reliability of concordance between independent observers

The kappa value was calculated to assess the concordance of the scale between observers. Also, the intraclass correlation (ICC) was calculated to assess the concordance between the observers. According to this analysis on concordance, the kappa value was in the range 0.80-0.97 before the operation, 0.63 - 0.99 during the operation and 0.85-0.97 after the operation (P < 0.001).

The intraclass correlation for the scale was found to be in the range 0.76-0.96 before the operation, 0.75-0.95 during the operation and 0.86-0.93 after the operation (P < 0.001) (**Table 5**). It was thus determined that there was good concordance between the observers.

**Table 5.** Intraclass correlation coefficients of the Neonatal Infant Acute Pain Assessment Scale (NIAPAS)

Characteristics	Intraclass correlation coefficient		
	Before operation	During operation	After operation
Gestational age	0.91	0.91	0.91
Alertness	0.88	0.93	0.88
Facial expressions	0.96	0.81	0.90
Crying	0.96	0.89	0.92
Muscle tension	0.91	0.93	0.87
Breathing	0.93	0.95	0.93
Reaction to handling	0.76	0.75	0.89
Heart rate	0.93	0.94	093
SaO_2_	0.94	0.95	0.86

Note: The intraclass correlation coefficient was calculated to assess the concordance between the observers. P < 0.001. SaO_2_ = Oxygen saturation.

## DISCUSSION

Pain is a subjective finding in newborns, who are unable to communicate verbally. Preterm and term newborns treated in NICUs are subject to various levels of painful interventions and this pain is an almost inevitable experience for such babies. Infants are known to feel pain and exhibit behavioral and psychological reactions to painful stimuli.^23^

Many scales are available to assess the pain of preterm and term newborns. Through having a valid, reliable and applicable measurement tool available, nurses will be better equipped to undertake pain management, thereby increasing the quality of patient care and avoiding pain for newborns who are particularly sensitive.

Unlike other scales, the NIAPAS scale assesses acute pain in newborns. Its most important distinction from other scales is that it was developed in close cooperation with specialist nurses in NICUs.^19^ Also, the NIAPAS scale enables both more detailed measurement of certain parameters and assessment of physiological changes in addition to behavioral patterns. This scale assesses acute pain through five behavioral and three physiological indicators that are exhibited as contextual factors in the gestational week at birth. It enables reasonable measurement of the pain felt by premature babies, through considering the gestational age in pain assessments on these infants.^19^^,^^24^ Reaside (2011) argued that oxygen saturation, blood pressure and respiration rate did not have any sensitivity or uniqueness and therefore could not be used independently.^25^ Holsti & Granau emphasized that independent measurement of behavioral and physiological reactions was necessary for assessing pain, in order to determine the effects of pain-relieving methods.^26^ Pölkki et al. emphasized that a multidimensional approach was the most suitable way for undertaking pain assessment.^19^

Our analysis on the validity of NIAPAS started with examination of its content validity. The SVI values of the scale items varied in the range 0.90-1.00. It has been stated in the literature that the situations that need to be measured using the items on the scale are well expressed by taking the criterion of an SVI value of 0.80.^27^

Concurrency validation is frequently performed in scale validity studies. It consists of assessing the concordance between one newly developed scale that has been cross-culturally adapted and another scale that was previously developed for the same purpose.^21^ Concurrency validation was performed in the present study between NIAPAS and NIPS. The correlation between the two scales was high before, during and after the operation. Moreover, there was a strong positive relationship between the correlation values of the two observers. The correlation found (**Table 2**) was close to the correlation values reported by Pölkki et al. (before the operation r = 0.751, during the operation r = 0.873 and after the operation r = 0.804).^19^

The correlation coefficient can range between +1 and -1. A (+) sign shows a positive and a (-) sign shows a negative relationship in the significance level.^28^ The correlation coefficient is zero if there is no relationship. It is accepted that the relationship is weak if the correlation coefficient is in the range 0.0-0.50, and strong if it is in the range 0.50-1.00.^29^ The concurrency validity of the NIAPAS scale was found to be high.

Cronbach’s alpha coefficient was calculated to assess the reliability of the scale. It was reported as 0.723 in the original NIAPAS study.^19^ In a study on the reliability of the NIAPAS scale, Huang et al. (2018) found that Cronbach’s alpha coefficient was 0.836.^30^ In the present study, the Cronbach’s alpha value was found to be 0.914, which is a higher value. Cronbach’s alpha coefficient indicates the internal consistency and the homogeneity of the items on a scale. According to the literature, the lower limit for Cronbach’s alpha coefficient was determined as 0.70 and it was stated that reliability increased as this number approached one.^31^ In the present study, Cronbach’s alpha coefficient for NIAPAS was found to be 0.914, thus showing that the scale was fairly reliable. This result was in line with data in the literature.^31^

Another method that has been used to test the reliability of a scale is item analysis.^21^ A correlation coefficient is calculated for item analysis. The effectiveness of the item increases with higher total item score correlation. Total item correlations are expected to be non-negative and a minimum of 0.20.^28^ The total item score correlations for NIAPAS were 0.20-0.82, which was in line with data in the literature,^28^ and no item needed to be removed from the scale.

In studies with multiple observers in which data are collected based on observations, concordance between the observers is one of the features that is required in determining the reliability of the scale.^22^ The kappa coefficient was used in the present study to test the concordance between the observers. This can range between 0 and +1, and negative values have no value in terms of reliability. The kappa coefficient indicates perfect concordance when it is in the range 0.93-1, very good concordance in the range 0.81-0.92 and good concordance in the range 0.61-0.80.^31^ The kappa value in the present study was > 0.73, according to the concordance analyses between the observers, and was therefore at the required level.

The intraclass correlation coefficient was also considered in assessing the concordance between the observers. The intraclass correlation needs to be at least 0.70 for any concordance between multiple assessors to be accepted.^32^ In the present study, the intraclass correlation was greater than 0.74, thus showing that there was concordance between the observers (**Table 5**). 

## CONCLUSION

Through the statistical analyses conducted to ascertain the validity and reliability of the Turkish form of the NIAPAS, it was determined that this scale is valid and reliable for use. 
